# British Medicinal Plants

**Published:** 1893-07

**Authors:** Alfred Heath


### BRITISH MEDICINAL PLANTS.

[Continued.]

ALFRED HEATH, M. D., F. L. S., ETC.

### ORDER 49.—LORANTHACEÆ.

*Viscum Album* (Mistletoe). This plant, the only one of the natural order Loranthaceæ found in Great Britain, is so well known to every one that we should hardly believe it was Christmas if a branch of mistletoe was not in the house. Hundreds of tons are annually collected in this country at Christmas. It is a true parasite, and is found on various trees in Great Britain, mostly on the apple tree. Rarely, if ever, is it found on the pear. It is also found on the poplar, hawthornes, limes, mountain ash, maples. It has been found on the cedar of Lebanon and on the larch, but rarely on the oak. Dr. Bull, in *Journal of Botany*, mentions only seven authentic instances of its occurrence on the oak. When growing on the oak it was worshiped with superstitious honors by the ancient Britons, to whom nothing was more sacred. The virtues of mistletoe grown on the oak are believed to be greater than if grown on any other tree. The Druidical priests (two thousand years ago) used to go attended with a procession of people clothed in white, and cut the mistletoe with a golden knife. Human sacrifices were offered on the spot; hymns sung and prayers recited in honor of the Divinity. The plant was then distributed amongst the people, which was considered a charm against every disease, and against evil spirits. The mistletoe has been highly extolled from the most ancient times for its medicinal virtues.

In times past the Viscum obtained great reputation in the cure of *epilepsy*, and a case is mentioned by Boyle, in which it proved remarkably successful in a lady of position. Some years after it was strongly recommended by Colbach in various kinds of *convulsions*. He published several instances of its good effects. He was supported by others who had used it with good effect in convulsions and other nervous diseases. The drug in these cases was given in a purely experimental manner and in total ignorance of the effects of the drug on healthy people, and of the fact that these cases were benefited or cured with a drug capable of producing on healthy person symptoms like epilepsy.

In Allen's *Materia Medica* is a short proving of Viscum, and it will be seen that it produced the following symptoms in healthy people: Two women took Viscum to procure abortion. Every muscle of the body except the eyes was *paralyzed;* the whole alimentary canal was paralyzed; they could neither speak or swallow, and both died in about eight or nine days—starved. It produces giddiness and slow, stertorous breathing, with drowsiness. A boy after eating eight berries had giddiness, conjunctivæ injected, pupil slightly dilated and fixed, lips livid, stertorous breathing, pulse full and bounding. A doctor took forty drops of the tincture; felt very queer as if he *must fall down; he felt a glow that rose up from the feet to the head*, and it seemed to him that he was on fire; at the same time his face became very pale. This kind of *aura epileptica* occurred three times during the winter (after forty drops). He felt on the back of the left hand *as if a large spider was crawling over it* (aura epileptica). Soon after he felt the same sensation on the back of the right hand (after forty drops).

If there is nothing in the "law of similars" how is it that a medicine like Viscum which obtained a reputation for curing epilepsy should in later times be found to *produce* on healthy people symptoms like that disease?

